# A Novel Method to Determine Basic Probability Assignment Based on Adaboost and Its Application in Classification

**DOI:** 10.3390/e23070812

**Published:** 2021-06-25

**Authors:** Wei Fu, Shuang Yu, Xin Wang

**Affiliations:** 1 Department of Automation, Heilongjiang University, Harbin 150080, China; 2191326@s.hlju.edu.cn (W.F.); 2201692@s.hlju.edu.cn (S.Y.); 2 Key Laboratory of Information Fusion Estimation and Detection in Heilongjiang Province, Harbin 150080, China

**Keywords:** Dempster-Shafer evidence theory, basic probability assignment, Adaboost, multiple strong classifiers, area ratio of the intersection region

## Abstract

In the framework of evidence theory, one of the open and crucial issues is how to determine the basic probability assignment (BPA), which is directly related to whether the decision result is correct. This paper proposes a novel method for obtaining BPA based on Adaboost. The method uses training data to generate multiple strong classifiers for each attribute model, which is used to determine the BPA of the singleton proposition since the weights of classification provide necessary information for fundamental hypotheses. The BPA of the composite proposition is quantified by calculating the area ratio of the singleton proposition’s intersection region. The recursive formula of the area ratio of the intersection region is proposed, which is very useful for computer calculation. Finally, BPAs are combined by Dempster’s rule of combination. Using the proposed method to classify the Iris dataset, the experiment concludes that the total recognition rate is 96.53% and the classification accuracy is 90% when the training percentage is 10%. For the other datasets, the experiment results also show that the proposed method is reasonable and effective, and the proposed method performs well in the case of insufficient samples.

## 1. Introduction

In the past decades, multi-sensor information fusion technology has received great attention and has been widely used in various fields, including military [1,2], medical [3,4], and financial [5] fields and so on [6,7]. In order to efficiently integrate information from different sources, it is crucial to choose an appropriate strategy. As one of the most important approaches in information fusion technology, DS evidence theory (DSET) has significant advantages. It can not only express uncertain information effectively but also fuse evidence without prior information [8,9,10]. Therefore, it has been used in a large variety of applications in various fields, including risk analysis [11], pattern recognition [12], fault diagnosis [13], and classification [14].

The effective application of Dempster’s combination rule relies on the rational construction of basic probability assignment (BPA, also called mass function). BPA is construction based on the identification data of multiple sensors and represents the support of one or more target classes. If BPA doesn’t reflect the characteristics of the target well, it may lead to counter-intuitive conclusions in the process of BPA combination, which is not the desired result. Therefore, in order to use DSET better, it is vital to find a reasonable way to determine BPA. In general, the determination of BPA can be divided into two types of methods. In the first type of method, BPA is determined by experts. Since the opinions of experts are subjective, the determined BPAs may sometimes be in great conflict. The other type is the data-driven method, in which the BPA is automatically determined in a certain way. Because of the complexity and diversity of the application background and the increasing demand for precision in evidence theory, the determination of the BPA in DSET remains a largely unsolved problem, to which there is no general solution. Many researchers have made attempts at this problem, which are generally based on two theories: probability distribution theory and fuzzy theory.

Under the condition of uniform distribution, BPA determination has been studied in [15]. In [16], the BPA determination is based on the assumption that the probability distribution of samples is the Gaussian model. In [17], BPAs are derived based on the probability distribution fitting of samples. The premise of most methods mentioned above is that the training samples have a specified distribution. However, the background of practical application is complex and diverse, and the specific distribution of subjective assumptions sometimes cannot meet the actual situation. Especially in the case of insufficient samples, it is even more difficult to determine the distribution of training samples. Therefore, once the assumed distribution is biased, it is difficult to get the desired result.

The methods based on fuzzy theory mainly use the triangular fuzzy number to determine BPA. For example, the extended triangular fuzzy number has been applied to determine BPA in the open world [18,19,20]. On the basis of [19,20], Xiao [21] proposed a BPA generation method, in which the k-means algorithm was used to optimize the BPA generation model. However, the construction of the membership function is a difficult and key point while using the fuzzy theory method to determine BPA. In particular, the membership function may be biased when the number of samples is small, which may decrease the accuracy and precision of BPA.

As a data-driven classification method, Adaboost has been widely used in many fields, including classification [22,23,24,25,26,27], fault diagnosis [28,29], pattern recognition [30,31,32], prediction [33,34,35,36], energy [37], aviation [38], and medical [39] fields. Adaboost does not make any assumptions about the probability distribution of samples. In addition, it has a simple structure and is not susceptible to overfit the training data [39]. Therefore, the characteristics of the Adaboost algorithm are very suitable for BPA determination.

Inspired by the above discussion, this paper proposes a novel BPA determination method without the assumptions about probability distribution for the sample, which is also effective when the number of samples is small. Based on the main steps of the Adaboost algorithm, the method determines the BPA by recording the weighted votes of the weak classifiers and using the area ratio method. The specific steps are as follows: First, several strong classifiers are generated by applying Adaboost. Second, the BPA of the singleton proposition is determined by weighted voting of the weak classifiers. Third, the proposed area ratio method is used to determine the BPA of the composite proposition. Finally, the final result is obtained by fusing all the BPAs using Dempster’s rule of combination.

The main contributions of this paper are as follows: (1) A novel method to determine BPA based on Adaboost is proposed, which is data-driven and does not make any assumptions about the probability distribution, so it can reduce the uncertainty of subjectivity. (2) The area ratio method is proposed to determine the BPA of the composite proposition, which improves the ability of BPA to deal with uncertain information. (3) The proposed method has a relatively high classification accuracy with a small number of training samples.

The structure of this article is organized as follows: Section 2 introduces the basic theories of DSET and Adaboost. Section 3 describes the proposed method and its architecture in detail. In Section 4, experiments are designed to elaborate on the effectiveness of the proposed method. At last, we summarize the results presented in this paper in Section 5.

## 2. Preliminaries

### 2.1. The Basic Theories of DSET

Compared with the probabilistic theory, DSET provides a powerful tool for the expression and combination of uncertainty information without prior probabilities, which makes it widely used in many data fusion systems. Some of its basic theories are as follows.

**Definition** **1.** ***Frame of discernment.***

*If a non-empty set* Θ *contains all the results of a target that people can identify, and the propositions contained in the set are mutually exclusive and exhaustive, then it is called the frame of discernment:*

(1) $$\Theta =\left\{\gamma_{1},\gamma_{2},\cdots ,\gamma_{n}\right\}$$

*where* $\gamma_{i}\left(i=1,2,\cdots ,n\right)$ *is the ith proposition of the discernment framework* Θ.

**Definition** **2.** ***Basic probability assignment***.

*Let* Θ *be the frame of discernment, then the function* $m:2^{\Theta }\to \left[0,1\right]$ *satisfies the following conditions:*

(2) $$m\left(\emptyset \right)=0$$

(3) $$\sum_{A\subset \Theta }m(A)=1$$

*where* m(A) *is called the BPA of* A *and it is understood to be the measure of the belief that is committed exactly to* A.

**Definition** **3.** ***Dempster’s rule of combination*.**

*Suppose that the two pieces of evidence* $E_{1}$ *and* $E_{2}$ *of the frame of discernment* Θ *have BPAs* $m_{1}$ *and* $m_{2}$ *and that* $A_{i}$ *and* $B_{j}$ *represent the different focal elements, respectively. Dempster’s rule of combination is then defined as follows [21]:*

(4) $$m(A)=m_{1}⊕m_{2}(A)=\left\{\begin{array}{c} 0\;A=\emptyset \\ \frac{\sum_{A_{i}\cap B_{j}=A}m_{1}(A_{i})\times m_{2}(B_{j})}{1-k}\;A\neq \emptyset \end{array}\right.$$

(5) $$k=\sum_{A_{i}\cap B_{j}=\emptyset }m_{1}(A_{i})\times m_{2}(B_{j})$$

*where* k *is the conflict coefficient and reflects the degree of conflict between the two pieces of evidence. It should be noted that Dempster’s rule of combination cannot be used when k=1, that is, when the two pieces of evidence are completely conflicting.*

### 2.2. Adaboost

Adaboost is a typical Boosting algorithm. In each iteration of training, Adaboost focuses more on misclassified samples and generates a relatively good model at the end. The algorithm not only has a simple structure but also has high accuracy [22,23,24,25,26,27,28,29,30,31,32,33,34,35,36,37,38,39]. Adaboost does not need to select attributes for training samples. Different classification algorithms can be used as weak classifiers and cascaded into strong classifiers. In practice, the simplest weak classifier is the decision stump, which is a form of decision tree with a single node and is commonly used in the framework of Boosting.

In this paper, the decision stump is used as the weak classifier. The two-classification process is taken as an example to specifically introduce the training process of the Adaboost:

**Step 1:** Choose the training set $D=\left\{\left(x_{1},y_{1}),\cdots ,(x_{i},y_{i}),(x_{N},y_{N}\right)\right\}$, where N is the number of the training samples, $x_{i}$ represents the *i*th sample of the training set, and $y_{i}\in \left\{-1,1\right\}$ is the class label of $x_{i}$.

**Step 2:** Initialize the weights of all training samples: $\omega_{1}(x_{i})=1/N$, where $\omega_{1}(x_{i})$ represents the weight of $x_{i}$ in the first iteration.

**Step 3:** Train the weak classifiers $h_{t}$. Let the maximum number of iterations be T (the number of weak classifiers), and then the training process of the *t*th (t∈[1,T]) iteration is as follows [39]:

(1) Train the weak classifiers $h_{t}$ by using $x_{i}$ and the weight $\omega_{1}(x_{i})$:

(6) $$h_{t}(x_{i})=\left\{\begin{array}{l} 1,\;\beta x_{i}(d)>\beta \theta_{t,d}\\ -1,\;\beta x_{i}(d)\leq \beta \theta_{t,d} \end{array}\right.$$

where $x_{i}(d)$ represents the *d*th attribute data of $x_{i}$, $\theta_{t,d}$ is the threshold value for the *d*th attribute in the *i*th iteration, and β∈{−1,1} is the direction of an attribute. It can be seen that the classification process of the decision stump is to compare the value of $x_{i}(d)$ with $\theta_{t,d}$. If $\beta x_{i}(d)$ is greater than $\beta \theta_{t,d}$, then the output is $h_{t}(x_{i})=1$ and $h_{t}(x_{i})=-1$, otherwise. β is used to correct the judgement logic when $h_{t}(x_{i})$ obtains counterintuitive conclusions.

(2) Calculate the error rate $\varepsilon_{t}$ of the weak classifiers for each attribute:

(7) $$\varepsilon_{t}=\frac{\sum_{i=1}^{N}L(y_{i},h_{t}(x_{i}))\omega_{t}(x_{i})}{\sum_{i=1}^{N}\omega_{t}(x_{i})}$$

where $L(y_{i},h_{t}(x_{i}))$ is zero-one loss function:

(8) $$L(y_{i},h_{t}(x_{i}))=\left\{\begin{array}{l} 1,\;y_{i}\neq h_{t}(x_{i})\\ 0,\;y_{i}=h_{t}(x_{i}) \end{array}\right.$$

(3) Calculate the weight $\alpha_{t}$ of the weak classifier:

(9) $$\alpha_{t}=\frac{1}{2}\ln\frac{1-\varepsilon_{t}}{\varepsilon_{t}}$$

(4) Update the weight distribution of samples:

(10) $$\omega_{t+1}(x)=\frac{\omega_{t}(x)\exp(-\alpha_{t}f(x)h_{t}(x))}{\sum_{i=1}^{N}\omega_{t}(x_{i})\exp(-\alpha_{t}f(x_{i})h_{t}(x_{i}))}$$

where the initial value $\omega_{1}(x_{i})=1/N$, x denotes any sample in $x_{i}$, and f(x) is the class label of the sample x.

**Step 4:** Obtain a strong classifier H by using Step 3 repeatedly:

(11) $$H(x)=\mathrm{sgn}(\sum_{t=1}^{T}\alpha_{t}h_{t}(x))$$

where sgn(x) is the sign function:

(12) $$\mathrm{sgn}(x)=\left\{\begin{array}{l} 1,\;x>0\\ 0,\;x=0\\ -1,\;x<0 \end{array}\right.$$

The calculation process of Adaboost is given in Algorithm 1.
**Algorithm 1.** The process of generating strong classifiers based on Adaboost**Input:** The training set: D, the number of classes in the dataset: $N_{c}$, the number of weak classifiers: T. **Output:** $C_{N_{c}}^{2}$ strong classifiers. 1: **For** *i* = 1: $N_{c}-1$ 2:   **For** *j* = *i* + 1: $N_{c}$ 3:     Attribute *i* and attribute *j* of the original training set are selected as the new training set $D_{i,j}$. 4:     The weight of the training sample is initialized to $\omega_{1}=1/N$. 5:      **For** *t* = 1: T 6:       Use the $\omega_{t}$ of $D_{i,j}$, the optimal weak classifier $h_{t}$ is trained from (6). 7:       Calculate the error rate $\varepsilon_{t}$ of $h_{t}$ from (7). 8:       Based on (9), calculate the weight $\alpha_{t}$ of $h_{t}$. 9:       Based on (10), the weight distribution $\omega_{t+1}$ of the sample is updated. 10:     **End For** 11:     Based on (11), a strong classifier $H_{i,j}$ belonging to attribute *i* and *j* is obtained. 12:   **End For** 13: **End For** 14: **Return** All the Strong classifiers $H=[H_{1,2},H_{1,3,},\cdots ,H_{N_{c}-1,N_{c}}]$.

**Figure 1:**
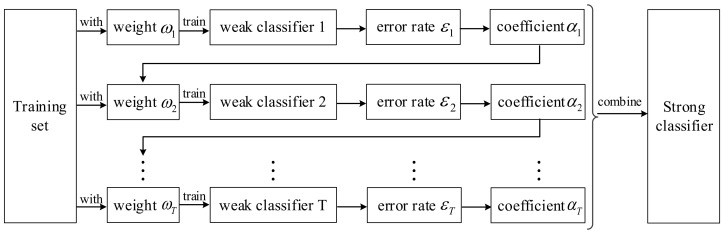
The flowchart of training a strong classifier in the Adaboost algorithm.

To further understand Algorithm 1, the computational flowchart of the algorithm is shown in Figure 1.

## 3. The Proposed Method for Determining BPA

In this section, we propose a novel method to determine BPA based on Adaboost. First, the process of determining the BPA of the singleton proposition is presented. Second, the area ratio method is proposed to obtain the BPA of the composite proposition.

### 3.1. Determine the BPA of the Singleton Proposition

Based on the Adaboost introduced in Section 2, the samples of any two attributes in the training set are taken as the new training set. It is assumed that the samples contain $N_{c}(N_{c}>1)$ classes, so we can get $C_{N_{c}}^{2}$ strong classifiers, where the *t*th ($t\in [1,C_{N_{c}}^{2}]$) strong classifier is composed of $T_{t}$ weak classifiers.

To better understand the process of the proposed method, we assume that A and B represent the first class and the second class of the dataset, respectively. If the *t*th strong classifier votes for the test sample x, then the voting result of the sample x belonging to class A is:

(13) $$m_{t}(A)=\sum_{k=1,h_{t,k}>0}^{T_{t}}\alpha_{t,k}/\sum_{k=1}^{T_{t}}\alpha_{t,k}$$

where $h_{t,k}$ is the $k\mathrm{th}(k=1,2,\cdots ,T_{t})$ weak classifier of the *t*th strong classifier and $\alpha_{t,k}$ is the weight of $h_{t,k}$. Because the class of sample x is either A or B in the current voting process, the voting result of the sample x belonging to class B is:

(14) $$m_{t}(B)=1-m_{t}(A)$$

When the voting results of all the strong classifiers in the current two attributes are recorded, the BPA of the test sample x belonging to class A is:

(15) $$m(A)=\frac{1}{C_{N_{c}}^{2}}\sum_{t=1}^{C_{N_{c}}^{2}}m_{t}(A)$$

The above voting results are determined based on any two attributes in the chosen samples. If there are n(n>1) attributes in the samples, $C_{n}^{2}$ BPAs can be determined. Similarly, the BPAs of other classes in the test samples can also be determined by using Equations (13)–(15).

The method in this section only determines the BPA of the singleton proposition, which is a lack of consideration for the uncertainty of the composite proposition. In order to improve the ability of the method to deal with uncertain information, we propose the area ratio method in Section 3.2.

### 3.2. Determine the BPA of the Composite Proposition

This paper proposes the area ratio method to reallocate the BPAs of samples located in the intersection region. The classification results for these samples are usually incorrect because it is difficult to distinguish which class they belong to, and this is a kind of uncertain information that needs to be expressed. Therefore, the area ratio method proposed in this section describes the intersection region of the sample distributions by constructing several rectangular regions. By calculating the area ratio between different regions, the mass of the singleton proposition is reallocated to the composite proposition.

For convenience, we introduce three notations:

(1) In the power set $2^{\Theta }$, any element whose cardinality is $1\leq l\leq N_{c}$ can be represented by the generic notation X(l). For example, in the frame of discernment Θ={A,B,C}, if the current two-classification process is to classify class A and class B, then X(1) denotes A or B, X(2) denotes the uncertainty AB, and X(3) denotes the uncertainty ABC.

(2) When l=1, area(X(l)) denotes the area of the rectangular region belonging to the samples of the singleton proposition. When l>1, area(X(l)) denotes the area of the intersecting rectangular region belonging to the samples of l different singleton propositions. For example, area(ABC) denotes the area of the intersection region of the rectangular regions A, B, and C.

(3) The area ratio of X(l−1) and X(l) is defined as follows:

(16) S(X(l),X(l−1))=area(X(l))/area(X(l−1))

In the process of BPA reallocation, the mass of the composite proposition is obtained by a recursive process. The mass m(X(l)) of uncertainty X(l) is obtained by the proportional reallocation of the mass m(X(l−1)), where $2\leq l\leq N_{c}$. The main process of recursive calculation is as follows:

(17) $$\left\{\begin{array}{l} m_{AR}(X(l))=\sum_{\begin{array}{l} X(l-1)\subset X(l)\\ X(l-1),X(l)\in 2^{\Theta } \end{array}}S(X(l),X(l-1))m_{AR}(X(l-1))\\ m_{AR}(X(l-1))=[1-S(X(l),X(l-1))]m_{AR}(X(l-1)) \end{array}\right.,2\leq l\leq N_{c}$$

with the initial value

(18) $$\left\{\begin{array}{l} m_{AR}(X(2))=\sum_{\begin{array}{l} X(1)\subset X(2)\\ X(1),X(2)\in 2^{\Theta } \end{array}}S(X(2),X(1))m(X(1))\\ m_{AR}(X(1))=[1-S(X(2),X(1))]m(X(1)) \end{array}\right.$$

Since the mass of the singleton proposition is reallocated to the uncertainty according to the area ratio, the mass of belief is not lost in the process of reallocation. The reallocated BPAs satisfy:

(19) $$\sum_{\begin{array}{l} X(l)\in 2^{\Theta }\\ 1\leq l\leq N_{c} \end{array}}m_{AR}(X(l))=1$$

**Figure 2:**
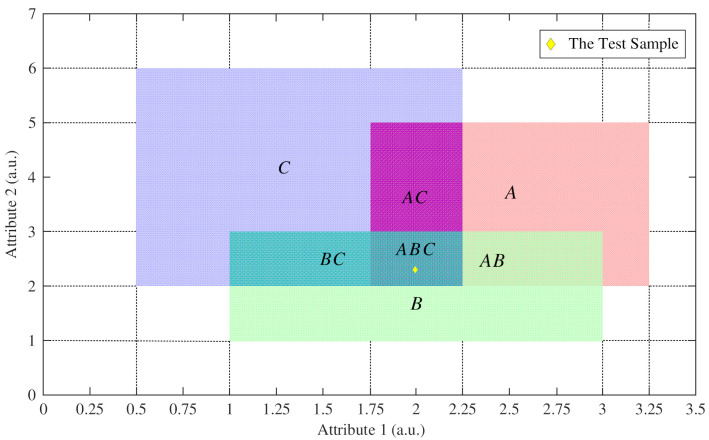
The rectangular distribution region of the three classes based on two attributes.

For example, let the frame of discernment be Θ={A,B,C} and consider that the sample regions of classes A, B, and C are shown in Figure 2. The areas of all rectangular regions are given in Table 1, where a.u. is short for the arbitrary unit.

If the mass of a sample is determined by the strong classifier of class A and class B, and the test sample is located in region ABC, as Figure 2 shows, the process of reallocating the mass of the singleton proposition according to the area ratio method is as follows:

(20) $$\left\{\begin{array}{l} S(AB,A)=area(AB)/area(A)=1.25/4.5=0.2777\\ S(AB,B)=area(AB)/area(B)=1.25/4=0.3125\\ S(ABC,AB)=area(ABC)/area(AB)=0.5/1.25=0.4 \end{array}\right.$$

(21) $$\left\{\begin{array}{l} m_{AR}(AB)=S(AB,A)m(A)+S(AB,B)m(B)=0.2777\times 0.6+0.3125\times 0.4=0.2916\\ m_{AR}(A)=(1-S(AB,A))m(A)=(1-0.2777)\times 0.6=0.4334\\ m_{AR}(B)=(1-S(AB,B))m(B)=(1-0.3125)\times 0.4=0.275 \end{array}\right.$$

(22) $$\left\{\begin{array}{l} m_{AR}(ABC)=S(ABC,AB)m_{AR}(AB)=0.4\times 0.2916=0.1166\\ m_{AR}(AB)=(1-S(ABC,AB))m_{AR}(AB)=(1-0.4)\times 0.2916=0.175 \end{array}\right.$$

The main process of determining BPA is shown in Algorithm 2.
**Algorithm 2.** The method to determine BPA**Input:** The training set: D, the number of classes in the dataset: $N_{c}$, the number of attributes in the dataset: n, the number of iterations: T. The test set P and the number of the test samples $T_{s}$. **Output:** BPAs of all samples in P. 1: **For** *i* = 1: n−1 2:   **For** *j* = *i* + 1: n 3:     Set the new training set $D_{i,j}=[D(:,i),D(:,j),y_{D_{i,j}}]$ based on the attribute *i* and *j* of D. 4:     Put $D_{i,j}$ into Algorithm 1 for training the $C_{N_{c}}^{2}$ strong classifiers $H_{i,j}$. 5:     Set the new test set $P_{i,j}=[P(:,i),P(:,j),y_{P_{i,j}}]$. 6:       **For** k = 1: $T_{s}$ 7:         **For** t = 1: $C_{N_{c}}^{2}$ 8:           $m_{t}(i)=\sum_{l=1,h_{l}(p_{i,j}(k))>0}^{T}\alpha_{l}/\sum_{l=1}^{T}\alpha_{l}$, where $h_{l}$ is the weak classifier of $H_{i,j}(t)$, $\alpha_{l}$ is the weight of $h_{l}$. 9:           $m_{t}(j)=1-m_{t}(i)$. 10:         **End For** 11:         $m_{p_{i,j}(k)}=\frac{1}{C_{N_{c}}^{2}}\sum_{t=1}^{C_{N_{c}}^{2}}m_{t}$. 12:         **If** $P_{i,j}(k)$ belongs to any intersection region 13:           Use (17) to reallocate the mass. 14:         **End If** 15:       **End For** 16:   **End For** 17: **End For** 18: Fuse all BPAs $m_{p_{i,j}(k)}$ using Dempster’s rule of combination to get the BPA $m_{p(k)}$ of P(k), $k=1,\cdots ,T_{s}$. 19: **Return** BPAs of all samples in P.

### 3.3. The Architecture of the Proposed Method

In this section, the process of BPA determination based on any two attributes is described in detail.

**Step 1:** Train the $C_{N_{c}}^{2}$ strong classifiers. The samples of any two attributes in the training set are taken as the new training set $D=\left\{\left(x_{1(1)},x_{1(2)},y_{1}),\cdots ,(x_{N(1)},x_{N(1)},y_{N}\right)\right\}$, where N is the number of the training samples, $x_{i(1)}$ and $x_{i(2)}$ represent the first and second attribute data of the *i*th sample in the training set, respectively, and $y_{i}\in \left\{1,2,\ldots ,N_{c}\right\}$ is the class label of $x_{i}$. The Adaboost algorithm in Section 2.2 is used to train $C_{N_{c}}^{2}$ strong classifiers, and the weights of all weak classifiers in the strong classifiers are recorded.

**Step 2:** Determine the BPAs by the trained classifiers. Similar to step 1, the samples of the same two attributes in the test set are taken as the new test set $P=\left\{\left(p_{1(1)},p_{1(2)},q_{1}),\cdots ,(p_{T_{s}(1)},p_{T_{s}(1)},q_{T_{s}}\right)\right\}$, where $T_{s}$ is the number of the test samples, $p_{i(1)}$ and $p_{i(2)}$ represent the first and second attribute data of the *i*th sample in the test set, respectively, and $q_{i}\in \left\{1,2,\ldots ,N_{c}\right\}$ is the class label of $p_{i}$. We use the classifier trained in step 1 to vote for each sample in the test set, then the BPAs of the test samples are determined by using Equations (13)–(15).

**Step 3:** Reallocate the BPA to express uncertainty. If the sample $\left(p_{i(1)},p_{i(2)}\right)$ is in the intersection region, then we use Equation (17) to reallocate the BPA determined in Step 2.

**Step 4:** Combination of BPAs. From Step 1 to Step 3, $C_{n}^{2}$ BPAs are determined for each test sample, and then we can use Dempster’s rule of combination to get the final BPA.

**Figure 3:**
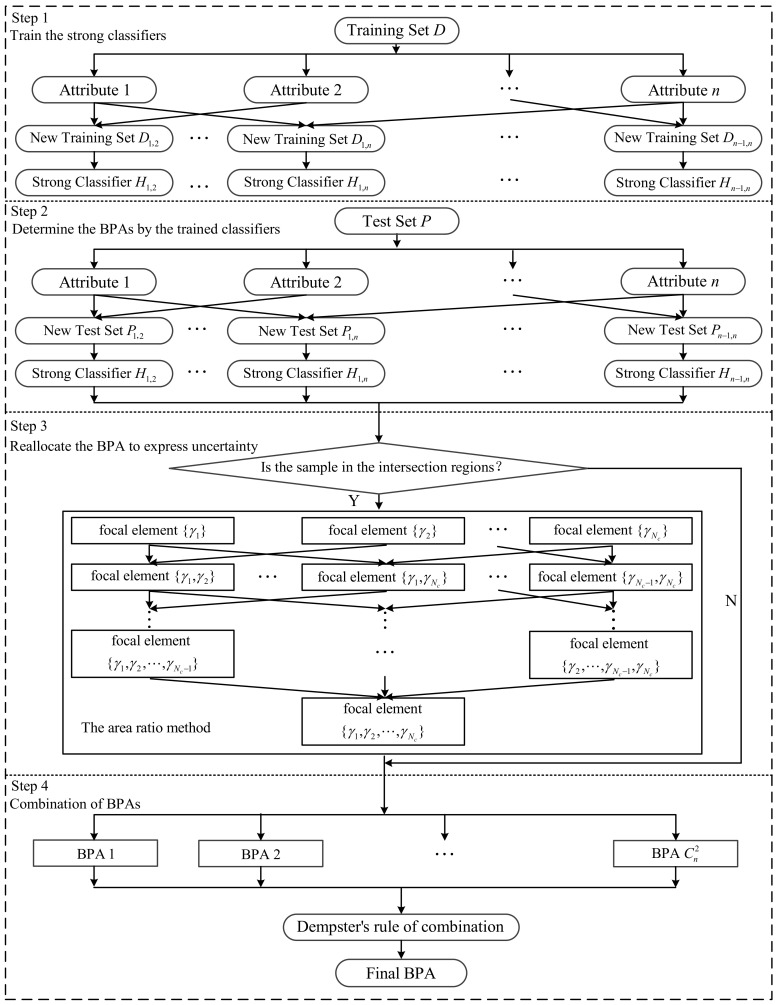
The flowchart of the four main steps in the proposed BPA determination method.

To better understand the proposed method, the flowchart of the proposed method is shown in Figure 3.

## 4. Experiments

In this section, we design some experiments to demonstrate the effectiveness of the proposed method in terms of classification and recognition by using the data from machine learning datasets. In Section 4.1, we show the proposed method with an example of determining BPAs by using the Iris dataset. In Section 4.2, we use four different datasets to test the classification accuracy and compare it with the classification accuracy of different methods.

### 4.1. An Example of Iris Dataset to Determine BPA

**Figure 4:**
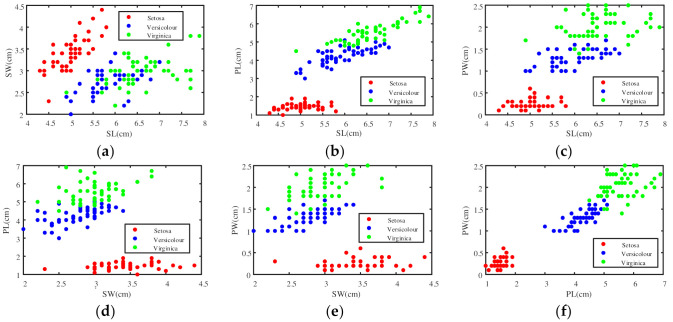
Six distribution figures based on any two attributes in the Iris dataset. (**a**) Sample distribution based on attributes SL and SW; (**b**) Sample distribution based on attributes SL and PL; (**c**) Sample distribution based on attributes SL and PW; (**d**) Sample distribution based on attributes SW and PL; (**e**) Sample distribution based on attributes SW and PW; (**f**) Sample distribution based on attributes PL and PW.

The Iris dataset is from the UC Irvine machine learning repository, which is one of the commonly used datasets in machine learning (http://archive.ics.uci.edu/ml/datasets/Iris) (accessed on 22 June 2021). The Iris dataset contains three classes: Setosa (Se), Versicolour (Ve), and Virginica (Vi). Each class contains 50 samples and has four attributes: sepal length (SL), sepal width (SW), petal length (PL), and petal width (PW). According to the proposed method in this paper, the four attributes can be used to determine six BPAs of a test sample. The sample distribution based on two attributes is shown in Figure 4.

#### 4.1.1. Determine the BPA of the Singleton Proposition

In this experiment, 40 groups of samples are randomly selected from each class of the Iris dataset, a total of 120 samples are selected as the training set, and the remaining 30 samples are used as the test set. According to the data of two attributes in the training set, a strong classifier is generated, which is used to vote the test samples to determine the BPA. The details of the experiment are shown below.

**Figure 5:**
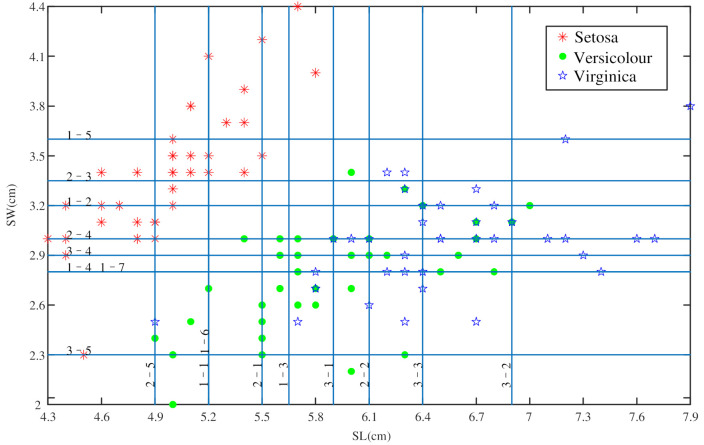
The process of using the weak classifiers to classify the samples based on SL and SW.

A sample from the test set of Virginica is taken as an example, and the data is given in Table 2. Since the training set contains four attributes, we can get six strong classifiers. Based on the training samples of SL and SW, Figure 5 shows the classification processes of the proposed method. Each line in the graph represents a weak classifier, and the meaning of the number above the line can be described as i−j, where *i* represents the *i*th two-classification process and *j* represents the *j*th weak classifier trained by the *i*th two-classification process.

**Figure 6:**
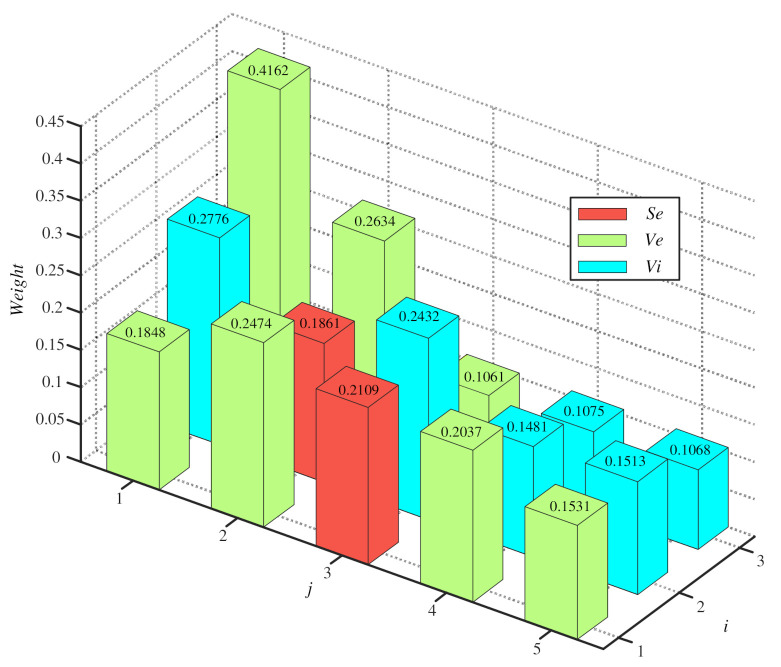
The weights of all weak classifiers based on Figure 5.

The weights of weak classifiers in different two-classifications are shown in Figure 6. Different colors represent different classes, and the heights represent the value of the votes. By using Equations (13)–(15) and the votes of all weak classifiers, the mass of this sample in SL and SW is given as follows:

m(Se)=0.1323, m(Ve)=0.5249, m(Vi)=0.3427

In the same way, we can also obtain the voting results of this sample in any other two attributes. However, the given sample of Virginica is located in the intersection regions of some sample distributions, and the uncertainty of the composite proposition should be considered. Therefore, we used the area ratio method in this example.

#### 4.1.2. Determine the BPA of the Composite Proposition

**Figure 7:**
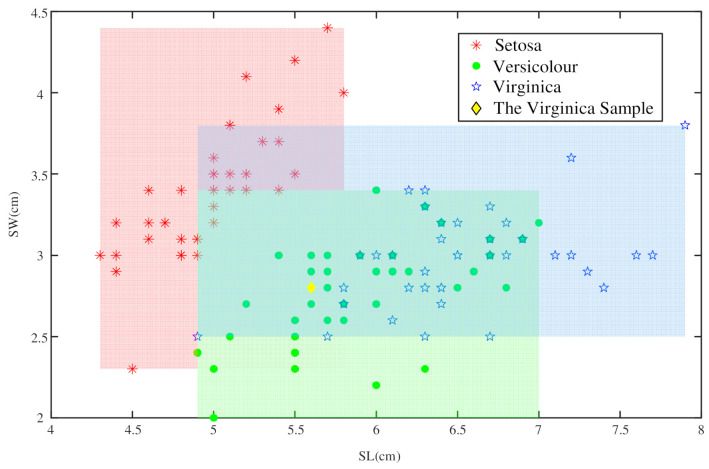
The sample distribution regions of the training set based on SL and SW.

As shown in Figure 7, we used rectangular regions with different colors to represent the distribution ranges of the sample distributions for the different classes of SL and SW. The given Virginica sample was located in the intersection region of three distribution regions. Therefore, we calculated the area ratios of the intersection regions to prepare for the reallocation of BPA. The ranges and the areas of all regions in this experiment are given in Table 3.

By using Equations (17) and (18), we get:

(23) $$\left\{\begin{array}{l} S(\{Se,Ve\},\{Se\})=area(\{Se,Ve\})/area(\{Se\})=0.3143\\ S(\{Se,Ve\},\{Ve\})=area(\{Se,Ve\})/area(\{Ve\})=0.3367\\ S(\{Se,Vi\},\{Se\})=area(\{Se,Vi\})/area(\{Se\})=0.3714\\ S(\{Se,Vi\},\{Vi\})=area(\{Se,Vi\})/area(\{Vi\})=0.3000\\ S(\{Ve,Vi\},\{Ve\})=area(\{Ve,Vi\})/area(\{Ve\})=0.6429\\ S(\{Ve,Vi\},\{Vi\})=area(\{Ve,Vi\})/area(\{Vi\})=0.4846 \end{array}\right.$$

(24) $$\left\{\begin{array}{l} S(\{Se,Ve,Vi\},\{Se,Ve\})=area(\{Se,Ve,Vi\})/area(\{Se,Ve\})=0.8182\\ S(\{Se,Ve,Vi\},\{Se,Vi\})=area(\{Se,Ve,Vi\})/area(\{Se,Vi\})=0.6923\\ S(\{Se,Ve,Vi\},\{Ve,Vi\})=area(\{Se,Ve,Vi\})/area(\{Ve,Vi\})=0.4286 \end{array}\right.$$

From Equations (23) and (24), we can reallocate the voting results of this sample of any two attributes. All the reallocated voting results and the result fused by Dempster’s rule of combination are given in Table 4.

From the values of the combined BPA in Table 4, we can conclude that the class of the test sample is Virginica, which is consistent with the result of the Iris dataset.

**Figure 8:**
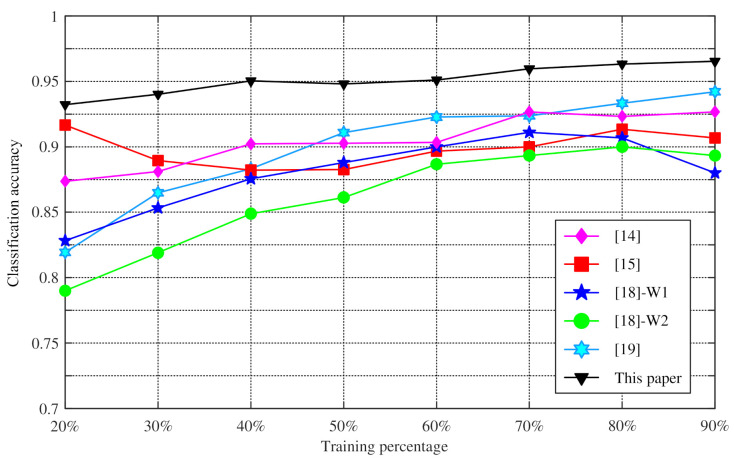
The accuracy comparison of four different methods with the method in this paper.

In order to demonstrate the superiority of the proposed method, we still take the Iris dataset as an example to compare the proposed method with the interval number method [15] and the generalized triangular fuzzy number method [14,18,19]. In this experiment, the number of training samples randomly selected from each class is 10, 15, 20, 25, 30, 35, 40, and 45. The remaining samples are used as the test set. The experiment is repeated 100 times using the Monte Carlo method, and the average value of the experimental results is recorded. As shown in Figure 8 and Table 5, the method proposed in this paper has higher classification accuracy.

### 4.2. Experiments on Changing the Training Percentage of Four UCI Datasets

In this section, we compare the proposed method with the following six well-known classifiers: support vector machine (SVM), SVM with radial basis function (RBF), RBF network (RBFN), multilayer perceptron (MP), naive Bayesian (NB), and Decision Tree learner (REPTree). We also consider the Adaboost mentioned in Section 2.2 to illustrate the effectiveness of the proposed method. In addition to the Iris dataset, the experiments in this section used three other datasets: Wine, Hepatitis, and Sonar, which are also from the UC Irvine machine learning repository.

The Wine dataset included 13 kinds of data, which were the result of chemical analysis of three different wines produced in the same region of Italy. The Hepatitis dataset contained 19 attributes, which included patient information and liver function test results, and the data of these attributes were used to predict whether a patient was alive or not. The Sonar dataset was used to predict whether the target object was a rock or a mine according to the strength data returned by a given sonar from different angles. The basic information about these datasets is given in Table 6, including the number of instances, the number of classes, the number of attributes, and the situation of missing values.

Table 7 shows the classification accuracy data of different classification methods using the above four datasets. In the experiment of each method, 80% of samples were randomly selected as the training set and the remaining samples as the test set. We then repeated the experiment 100 times and used the average accuracy of these experiments as the final accuracy. By comparing the average accuracy of each method, it follows that the proposed method in this paper is more effective.

**Figure 9:**
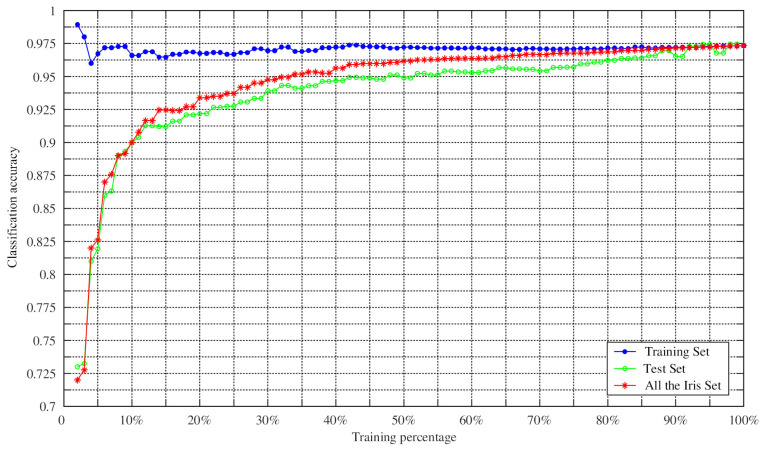
Classification accuracy versus training percentage for the Iris datasets.

**Figure 10:**
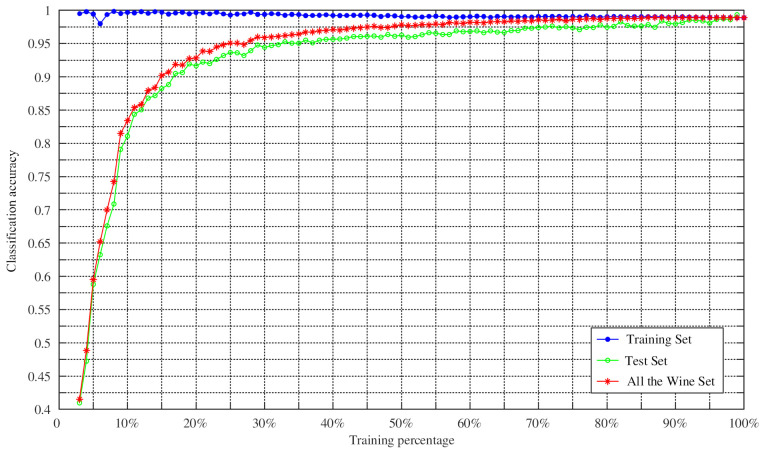
Classification accuracy versus training percentage for the Wine datasets.

**Figure 11:**
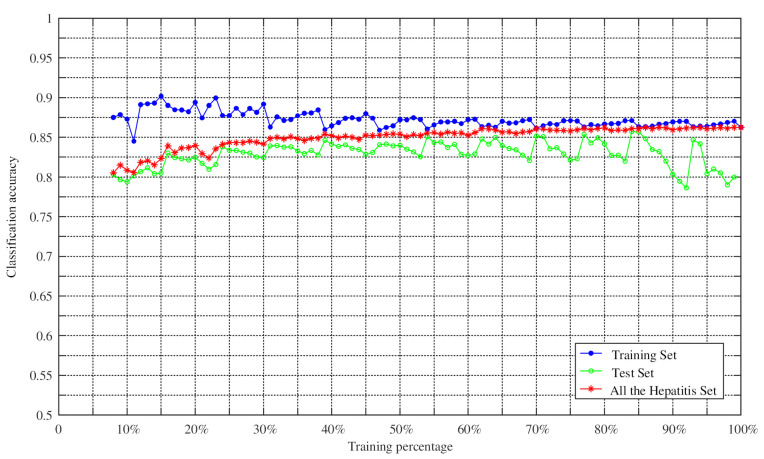
Classification accuracy versus training percentage for the Hepatitis datasets.

**Figure 12:**
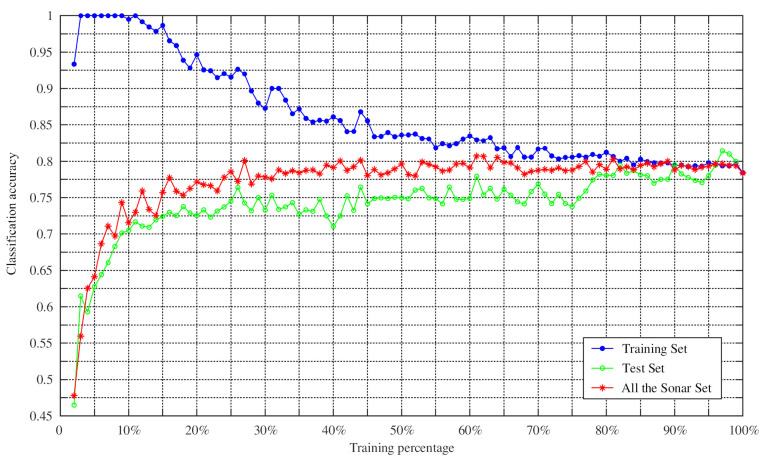
Classification accuracy versus training percentage for the Sonar datasets.

To verify the effectiveness of the proposed method in classification, the proposed method was further tested by changing the training percentage. N percent of the dataset samples were randomly selected as the training set, and the remaining samples were used as the test set. We set the training percentages of the Hepatitis dataset from 8% to 98% because it contained missing values, while the training percentages of other datasets changed from 2% to 98% during the training process. The Monte Carlo method was then employed to repeat the experiment 100 times to obtain the average classification accuracy of the training set, the average classification accuracy of the test set, and the average classification accuracy of the whole dataset. The experimental results are shown in Figure 9, Figure 10, Figure 11 and Figure 12.

As can be seen from Figure 9, Figure 10, Figure 11 and Figure 12, the average classification accuracy for the Iris dataset, the Wine dataset, and the Sonar dataset improved with the increasing training percentage. However, for the Hepatitis dataset, the trend of the average classification accuracy was not similar to the others and decreased as the number of the test samples increased. This is because there were 60 attributes in the Hepatitis dataset and the area of the intersection region between different attributes was large. This situation increased the difficulty of classification, which is the reason why most algorithms have similar classification accuracies in the Sonar dataset. Nevertheless, the average classification accuracy of the Hepatitis dataset was still relatively high.

In addition, in the field of practical application, a large number of training samples may not be obtained. Therefore, in this case, the feasibility of the method for determining BPA was particularly important. As can be seen from Table 8, the accuracies of Iris dataset and Sonar dataset reached 81.28% and 90.26%, with a training set of 10%, respectively. When the training percentage was 15%, the accuracies of Wine dataset and Hepatitis dataset were 88.2% and 80.5%, respectively. It is worth noting that the average classification accuracy of the four datasets was 80.25% when the training proportion was 10%. These results show that the method in this paper was still reasonable and effective in the case of a small number of training samples.

## 5. Conclusions

In Dempster-Shafer evidence theory (DSET), how to determine a reasonable basic probability assignment (BPA), which is a crucial and the first step, is still an open issue. In this paper, a novel method to determine BPA based on Adaboost is proposed. In this proposed method, multiple strong classifiers were constructed using the training samples and the corresponding weights were recorded, which were used to determine the BPA of the singleton proposition. The BPA of the composite proposition was determined by the area ratio of the intersection region of the singleton proposition. The advantages of the proposed method are as follows:

1. The proposed method in this paper is data-driven so that the uncertainty caused by subjectivity is reduced.
2. No assumption is made about the training data distribution, which allows the method to be applied in many different fields.
3. The area ratio method is proposed to improve the ability of BPA to deal with uncertain information and increase the accuracy and precision of classification.
4. The method is simple and practical and it can determine BPA in the case of a small number of training samples. Using the proposed method to classify the Iris dataset, the experiment concludes that the total recognition rate is 96.53% and the average classification accuracy of 90% can be reached when the training percentage is 10%.

When there are too many attributes of the training sample, it will cause a larger computational burden, which is the limitation of this paper. As an extension of the results of this paper, the BPA determination methods based on multi-attribute classification will be considered in our future work.

## Figures and Tables

**Table 1:** The areas of all rectangular regions.

Proposition	*A*	*B*	*C*	*AB*	*AC*	*BC*	*ABC*
Area	4.5	4	7	1.25	1.5	1.25	0.5

**Table 2:** The attribute’s value of the sample.

Attribute	SL	SW	PL	PW
Value	5.6	2.8	4.9	2

**Table 3:** The range and the areas of all regions (cm).

	$X_{\min}$	$X_{\max}$	$Y_{\min}$	$Y_{\max}$	Area
{Se}	4.3	5.8	2.3	4.4	3.15
{Ve}	4.9	7.0	2.0	3.4	2.94
{Vi}	4.9	7.9	2.5	3.8	3.90
{Se,Ve}	4.9	5.8	2.3	3.4	0.99
{Se,Vi}	4.9	5.8	2.5	3.8	1.17
{Ve,Vi}	4.9	7.0	2.5	3.4	1.89
{Se,Ve,Vi}	4.9	5.8	2.5	3.4	0.81

**Table 4:** All the determined BPAs and the combined BPA.

Attributes	m(Se)	m(Ve)	m(Vi)	m(Se,Ve)	m(Se,Vi)	m(Ve,Vi)	m(Se,Ve,Vi)
SL,SW	0.0872	0.2680	0.2267	0.0201	0.0321	0.1160	0.2498
SL,PL	0	0.4195	0.5035	0	0	0.0770	0
SL,PW	0	0.3533	0.6467	0	0	0	0
SW,PL	0	0.4331	0.5027	0	0	0.0642	0
SW,PW	0	0.3333	0.6667	0	0	0	0
PL,PW	0	0.3631	0.6369	0	0	0	0
Combined BPA	0	0.1088	0.8912	0	0	0	0

**Table 5:** Accuracy versus training percentage for different methods.

Percentage	[14]	[15]	[18]-W1	[18]-W2	[19]	This Paper
20%	87.37%	91.67%	82.83%	79.00%	81.93%	93.23%
30%	88.10%	88.95%	85.33%	81.90%	86.49%	94.02%
40%	90.22%	88.22%	87.56%	84.89%	88.33%	95.04%
50%	90.27%	88.27%	88.80%	86.13%	91.09%	94.80%
60%	90.33%	89.67%	90.00%	88.67%	92.27%	95.10%
70%	92.67%	90.00%	91.11%	89.33%	92.36%	95.96%
80%	92.33%	91.33%	90.67%	90.00%	93.33%	96.33%
90%	92.67%	90.67%	88.00%	89.33%	94.20%	96.53%

**Table 6:** The information of different datasets.

Dataset	Instance	Class	Attribute	Missing Value
Iris	150	3	4	No
Wine	178	3	13	No
Hepatitis	155	2	19	Yes
Sonar	208	2	60	No

**Table 7:** The accuracy of different classifiers (%).

Data	SVM-RBF	REPTree	MP	NB	SVM	RBFN	Adaboost	This Paper
Iris	92.7	94.7	96	96	96.7	96.7	93.6	96.2
Wine	39.9	93.3	95.5	95.5	94.4	97.2	96.1	97.4
Hepatitis	79.4	79.4	79.4	84.5	81.9	84.5	84.1	84.2
Sonar	74.4	75.5	80.8	69.2	78.8	75.5	74.8	78.0
Average	71.60	85.73	87.93	86.30	87.95	88.48	87.15	88.95

**Table 8:** Accuracy versus training percentage for different datasets.

Proportion	Iris	Wine	Hepatitis	Sonar	Average
10%	90.0%	81.1%	79.4%	70.5%	80.25%
15%	91.2%	88.2%	80.5%	72.4%	83.08%
20%	92.2%	91.7%	82.5%	72.5%	84.73%
25%	92.7%	93.6%	83.3%	74.5%	86.03%
30%	93.9%	94.4%	82.4%	73.3%	86.00%
35%	94.1%	95.1%	83.3%	72.7%	86.30%
40%	94.7%	95.7%	84.1%	71.0%	86.38%
45%	94.9%	96.1%	82.8%	74.1%	86.98%
50%	94.9%	96.3%	84.0%	75.0%	87.55%
55%	95.1%	96.6%	84.3%	74.9%	87.73%
60%	95.3%	96.8%	82.7%	74.9%	87.43%
65%	95.7%	96.7%	83.9%	76.2%	88.13%
70%	95.4%	97.4%	85.1%	76.8%	88.68%
75%	95.7%	97.4%	82.1%	73.8%	87.25%
80%	96.2%	97.4%	84.2%	78.0%	88.95%
85%	96.4%	97.6%	85.7%	78.1%	89.45%
90%	96.5%	98.0%	80.3%	78.8%	88.40%
95%	97.4%	98.1%	80.4%	78.0%	88.48%
